# Extreme oncoplasty for centrally located breast cancer in small non-ptotic breasts: extending the indications of chest wall perforator flaps with areolar reconstruction

**DOI:** 10.3332/ecancer.2021.1311

**Published:** 2021-11-01

**Authors:** Shashank Nigam, Andrew Eichholz, Madhu Bhattacharyya, Vaishali Parulekar, Pankaj Gupta Roy

**Affiliations:** 1 Department of Breast Surgery, Oxford University Hospitals NHS Foundation Trust, Oxfordshire OX3 7LF, UK; 2 Private Oncology Clinic, Lucknow, UP 226021, India; 3 Department of Oncology, Oxford University Hospitals NHS Foundation Trust, Oxfordshire OX3 7LF, UK; 4 Oxford Breast Imaging Centre, Oxford University Hospitals NHS Foundation Trust, Oxfordshire OX3 7LF, UK

**Keywords:** chest wall perforator flap, partial breast reconstruction, central excision, volume replacement oncoplastic breast surgery, centrally located breast cancer, extreme oncoplasty

## Abstract

**Background:**

Breast cancers located centrally require excision of nipple-areola complex. A simple central wide excision is a safe option but results in suboptimal aesthetic outcome. An oncoplastic option involves therapeutic mammoplasty with or without areolar reconstruction, limited to moderate and large ptotic breasts. For small non-ptotic breasts, most surgeons would resort to mastectomy with/without reconstruction.

**Methods:**

Lateral chest wall perforator flap (CWPF) is an option for partial breast reconstruction in small to moderate sized, non-ptotic breasts for laterally located tumours. We have extended the application of CWPF for central tumours to avoid mastectomy in selected patients.

**Results:**

We here present a case series of four patients with small to medium-sized non-ptotic breasts, who had centrally located breast cancer or ductal carcinoma in-situ (DCIS). Three patients had single stage CWPF reconstruction, and one had central excision with immediate reconstruction following a failed attempt at therapeutic mammoplasty. All had the areola reconstructed using flap skin; one patient had simultaneous nipple reconstruction.

**Conclusions:**

CWPF is an option for treatment of centrally located breast cancers/DCIS needing nipple-areola complex excision for patients wishing to avoid mastectomy. Patients with small to medium-sized non-ptotic breasts are suitable, and need to be carefully selected.

## Introduction

Breast conserving surgery (BCS) with adjuvant breast radiotherapy is the standard of care for early breast cancer. Trials have shown equivalence of this treatment to mastectomy with respect to survival and local disease control [1–5]. It leads to improved quality of life, high patient satisfaction and good cosmetic results [6, 7]. Indications for BCS have gradually been extended for larger tumour to breast ratio, or for tumours located at difficult sites by incorporating plastic surgery techniques to perform either volume displacement or replacement surgery [8, 9]. Oncoplastic breast surgery (OBS) has been found to be oncologically safe both for invasive and in-situ carcinoma, and results in better aesthetic and psychological outcomes [10–14]. Tumour location is an important factor influencing choice of oncoplastic technique.

Between 5% and 20% of breast cancers are located centrally [15–18]. Historically these have been treated with mastectomy. The oncological safety of BCS to treat centrally located breast cancer has now been established [17, 19–21]. Centrally located breast cancer requires excision of the nipple-areola complex for oncological reasons [22]. A simple central wide excision with removal of the nipple-areola complex is oncologically safe and adequate (once complete resection is established histologically), however it results in residual breast which aesthetically looks suboptimal [23, 24].

Therapeutic mammoplasty with or without areolar reconstruction has been used to treat centrally located breast cancers with good outcomes [24–33]. It is usually indicated for moderate to large ptotic breasts and often necessitates contralateral symmetrisation surgery [12, 34]. For small to moderate sized non-ptotic breasts presenting with centrally located breast cancer, this option is limited and most surgeons would resort to mastectomy with or without reconstruction.

The evolution of OBS has seen various surgical techniques being employed for partial breast reconstruction in an attempt to improve the aesthetic outcomes and reduce mastectomy rates. Volume replacement using chest wall perforator flap (CWPF) is one such procedure, initially used by Holmström and Lossing* et al* [35], and later popularised by Hamdi *et al* [36–38]. These flaps are based on perforators supplying the lateral chest wall; namely lateral intercostal artery perforators (LICAP), lateral thoracic artery perforator (LTAP) and thoraco-dorsal artery perforators. This approach utilises the redundant lateral chest wall fold for reconstructing the partial breast defect resulting from excision of laterally placed tumours in small to moderate sized non-ptotic breasts. With increasing experience, surgeons have extended its indications. It is associated with minimal procedure-related morbidity, quick recovery and excellent aesthetic outcomes [39, 40]. The surgical technique and pre-operative marking are detailed in the previously published paper [41].

### Objectives

We report challenging cases of centrally located breast cancers with nipple involvement or proximity to nipple necessitating excision of nipple-areola complex. The defect after wide local excision was reconstructed using the tissue from the CWPF and areola was reconstructed using the flap skin.

## Materials and methods

These women had small to moderate sized non-ptotic breasts with relatively high tumour to breast ratio, as judged by imaging (multifocal cancer or extensive ductal carcinoma in-situ (DCIS)). All these women were keen to avoid mastectomy, thus partial breast reconstruction was performed using LICAP and/or LTAP based flap. The CWPF was tunnelled deep to the native breast tissue on the lateral aspect and then folded to ensure adequate volume to fill the defect, such that skin was oriented anteriorly, after folding, to reconstruct the areola. The resultant tunnelling led to fullness on the lateral aspect of breast but that did not seem to be of significant concern and did not result in significant asymmetry after radiotherapy. The aesthetic outcome was assessed by the clinical team using Harris scale (scale from 1 to 5) in the out-patient clinic between 1 and 3 years post-radiotherapy. All these women had ER positive, Her-2 negative breast cancer and received adjuvant treatment as per Multidisciplinary Team discussion. These cases took between 2 and 3 hours to operate and patients were discharged home the day after surgery. These patients encountered no significant complications, and no delay in adjuvant therapy was observed. One patient required margin re-excision and all patients received adjuvant radiotherapy. All patients have completed at least 1-year follow-up and are happy with the aesthetic outcome.

## Results (clinical and treatment details)

**Case 1:** This lady was identified to have bifocal breast cancer in her left breast on her first screening mammogram at the age of 50. She was on hormone replacement therapy. There were two areas, one measuring 24 mm just lateral to the left nipple (with nipple tethering), grade I invasive cancer on core biopsy. The second lesion was 15 mm in the upper outer quadrant (17 mm away from the first lesion), confirmed to be grade II invasive cancer on biopsy. The total extent was 54 mm in the cranio-caudal plane and 30 mm in the medio-lateral plane. Both the tumours were oestrogen receptor (ER) 8, progesterone receptor (PR) 8 and human epidermal growth factor receptor (Her-2) negative.

The patient had symmetrical breasts with a bra size of 32B. There was a prominent fold of tissue excess on the lateral chest wall, rendering CWPF as a potential option. She was offered mastectomy with immediate reconstruction, as the total size of the two cancers together made BCS a borderline option. The patient preferred to have breast conservation; therefore, staged approach to partial breast reconstruction was discussed. Patient chose to have one stage approach after careful deliberation, accepting the option of simple mastectomy if BCS was not successful. She had central wide excision and sentinel node biopsy with single stage partial breast reconstruction using LICAP flap with neo-areolar inset using the skin from the lateral chest wall.

Histopathology confirmed bifocal invasive grade 2 cancer measuring 16 and 25 mm, with intermediate grade (IG) DCIS. The whole tumour size was 60 mm, and was excised with clear margins (superior margin being closest, 1 mm away). The sentinel node removed was positive for macrometastasis (6 mm tumour deposit) with no extra-capsular spread. Histopathological examination of the nipple showed DCIS 2 mm from the skin surface. She was recommended adjuvant chemotherapy, radiotherapy to breast (with tumour bed boost), axilla and supra-clavicular fossa along with endocrine therapy. She consented to enter the POSNOC trial [42] and was randomised to systemic therapy alone with no further local treatment to the axilla. The post-operative period was uneventful with no complications. The patient was very pleased with the outcome. She continues on Anastrozole with good tolerance.

**Case 2:** A 72-year-old lady presented symptomatically with left sub-areolar lump. This was occult on mammogram and ultrasound. A clinical core biopsy confirmed grade 2 invasive lobular carcinoma (ILC). The tumour was ER8, PR8 and Her-2 negative. MRI of breasts suggested a unifocal lesion measuring 26 mm. She was fit and well with an active lifestyle. She declined the option of central wide local excision with therapeutic mammoplasty, as she did not wish asymmetry or need for external prosthesis and was keen to avoid contralateral breast surgery. She agreed to central wide local excision and immediate partial breast reconstruction with CWPF with sentinel node biopsy. She underwent simultaneous nipple reconstruction.

Histopathology revealed bifocal grade 2 invasive lobular cancers measuring 26 and 8 mm within high grade (HG) DCIS, total extent of DCIS was 50 mm. The inferior margin was focally involved with DCIS. One out of two sentinel nodes showed evidence of macrometastasis (10 mm tumour deposit, without extra-capsular spread). The inferior margin was re-excised a few weeks later; access was gained via peri-areolar scar. No further disease was seen on histology. Her recovery was uneventful and there were no complications with full healing of the reconstructed nipple. She declined participation in the POSNOC trial [42] hence received treatment to axilla in line with standard treatment protocols in our centre. It was decided to avoid axillary node clearance in favour of axillary radiotherapy.

**Figure 1.**
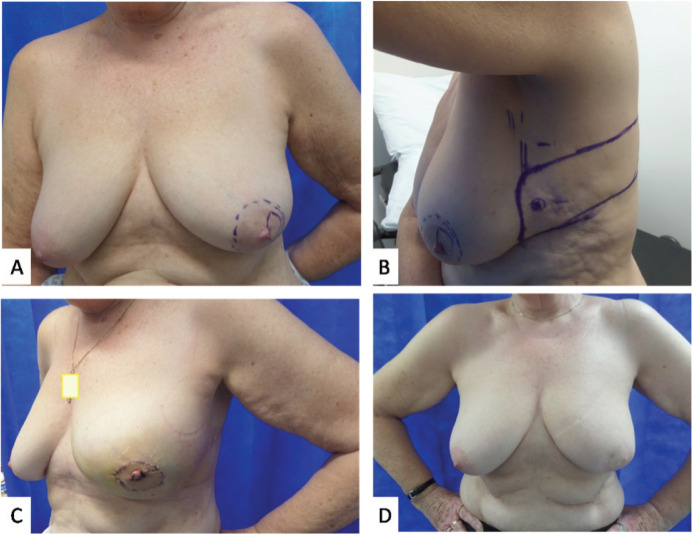
Photographs of case 2. (a): Marked sub-areolar lump with dashed markings for proposed central excision. (b): Lateral view showing the markings of CWPF with cross marked at perforator as identified using a handheld Doppler. (c): 2 weeks post-operative results showing reconstructed central defect with nipple and areolar reconstruction from skin of the flap. (d): 1 year post surgery and post radiotherapy results.

Breast multidisciplinary team recommended adjuvant radiotherapy to breast, axilla and supraclavicular fossa (SCF) with endocrine therapy. The National Health Service (NHS) PREDICT suggested 4% survival benefit with third-generation chemotherapy at 10 years, therefore OncotypeDX testing was recommended, which reported a low recurrence score of 11, suggesting very small benefit from chemotherapy. She was advised Anastrozole as endocrine therapy. She was reviewed in clinic a year after radiotherapy and has achieved an excellent aesthetic outcome with regard to size/shape of breast and symmetry (Figure 1).

**Case 3:** A 40-year-old lady was evaluated in a symptomatic clinic for a lump corresponding to a 35 mm mass on mammogram without calcifications in the lower outer quadrant of breast. This mass measured 22 mm on ultrasound, while axillary nodes were normal. Core biopsy confirmed grade 3 invasive ductal carcinoma (IDC) with HG DCIS. Receptor profile was ER8, PR6 and Her-2 negative. This lady had difficult family circumstances and was caring for a disabled family member. She was offered breast conservation with vertical scar therapeutic mammoplasty and sentinel node biopsy.

The histopathology showed 35 mm of invasive ductal cancer associated with extensive HG DCIS with a total tumour extent of 60 mm. Lymphovascular invasion was present. A separate medial shave sent during surgery showed the presence of DCIS within 1 mm from the final margin. The single sentinel node removed showed micrometastasis with a tumour deposit of 1 mm. As the medial margin was the margin adjacent to nipple, re-excision of this margin mandated a central nipple-areola excision. She was, therefore, offered mastectomy with reconstructive options due to presence of extensive, non-calcified DCIS, the extent of which was impossible to estimate with the available imaging.

**Figure 2.**
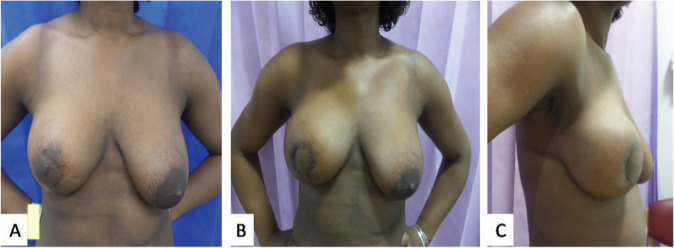
Photographs of case 3. (a): Approximately 6 months following a right vertical scar therapeutic mammoplasty for tumour located in the lower outer quadrant. The total tumour extent was 60 mm with DCIS close to medial margin. She completed adjuvant chemotherapy with a plan for mastectomy thereafter. As she was still keen on conserving her breasts, a central wide excision and partial breast reconstruction using CWPF were performed. (b and c): Front & lateral view of 3 weeks post-operative results showing reconstructed central defect with areolar reconstruction from skin of the flap.

A decision was made in favour of adjuvant chemotherapy with a plan to do a completion mastectomy following chemotherapy. She was not keen for autologous reconstruction due to the extent of surgery and the recovery period involved. Implant reconstruction was considered suboptimal in light of the recommendation for adjuvant radiotherapy. The patient was very keen to consider the possibility of BCS. Upon reviewing her surgical options post chemotherapy and noting the limited choices available, she was offered an attempt at breast conservation by performing a central excision and partial breast reconstruction with a CWPF and neo-areolar inset. She understood that if this were to fail on account of finding more extensive disease, she would be recommended mastectomy. She underwent the proposed procedure and the histology did not reveal any residual malignancy. Following second surgery, her affected breast looked bigger than the opposite breast, this settled following radiotherapy to the whole breast with a boost to the tumour bed. She continues on Tamoxifen and is very pleased with the aesthetic outcome achieved (Figure 2).

**Case 4:** A 36-year-old lady was assessed in a symptomatic breast clinic for unilateral single duct nipple discharge. Ultrasound did not reveal any focal pathology, while the nipple discharge cytology showed epithelial cells suggesting an epithelial lesion. She had total duct excision. Histology revealed 6 mm of grade1 incidental mucinous carcinoma with 17 mm of HG DCIS, with DCIS involving the margins. The tumour was ER 8, PR 5 and Her-2 negative. She was recommended therapeutic surgery.

She was slim and small breasted with a bra cup of AA, but there was some redundant tissue on the lateral chest wall. Patient declined mastectomy, therefore BCS with partial breast reconstruction was offered as a potential alternative.

She underwent central wide local excision and sentinel node biopsy with partial breast reconstruction using a CWPF with neo-areolar inset. Central excision specimen showed a further 3 mm of grade 2 mucinous carcinoma with HG DCIS. This was excised with clear margins, with the closest peripheral margin being more than 10 mm. She made a good recovery and received adjuvant whole breast radiotherapy. She continues on Tamoxifen and is very pleased with the aesthetic outcome.

The clinico-pathological and treatment details of these patients have been summarised in Tables 1 and 2, respectively.

## Discussion

Centrally located cancers have traditionally been treated with mastectomy; however, in the era of oncoplastic surgery, it would be inappropriate to offer mastectomy purely due to location of tumour. Small central tumours could be managed with simple central wide local excision and primary closure. Various closure techniques have been attempted including purse-string, vertical or horizontal closure [24, 43]. This often leads to sub-optimal cosmetic results due to loss of breast volume and projection, although these options may be acceptable to some patients. Therefore, it is reasonable to offer the simple option to patients not wishing to undergo complex surgery or who are otherwise high risk for anaesthesia.

Volume displacement oncoplastic techniques advocated for central cancers include Grisotti’s infero-lateral dermo-glandular flap, mammoplasty with inferior dermo-glandular flap or melon-slice mammoplasty. These techniques either rely on creating neo-areola from patch of skin located inferior/infero-lateral to nipple-areola, or simply closing the breast in a way that maintains the shape of breast [16, 25, 26, 29, 32, 44, 45]. Galimberti* et al* [30] first described the infero-lateral dermo-glandular flap, which was later popularised by Grisotti* et al* [24]. However, most of these techniques are applicable mostly to moderate or large ptotic breasts. These procedures often necessitate contralateral symmetrisation surgery.

Volume replacement technique for central tumours described in the literature involves partial breast reconstruction using a latissimus dorsi flap [26, 46–51], which adds significant muscle morbidity. Other techniques of volume replacement like use of omental flap [52] or free dermal fat graft [43] have not been easy to reproduce and can have a significant risk of complications.

CWPF has been increasingly used in the last decade for treatment of laterally placed breast cancers in small to moderate sized non-ptotic breasts [36]. They have been shown to be oncologically safe with low morbidity and good recovery of shoulder function and do not involve muscle morbidity [36, 39, 53]. When compared with mastectomy and reconstruction, CWPF offers quicker recovery, lower complication rates and better aesthetic results [38, 39, 41]. With increasing experience, longer flaps can be reliably raised based on LICAP and LTAP to reach central quadrants defects for breast cancer management. The skin inset to create areola is usually from the distal half of the flap providing an option to monitor flap vascularity in the post-op period, helping to improve operator’s confidence.

It has been shown that these flaps do not interfere with radiological surveillance, and recall rates for biopsy are low [54, 55]. The flap is tunnelled into the defect behind the breast tissue, ensuring that residual breast tissue sits in front of the flap, thus avoiding the potential hindrance from the flap to allow detection of local recurrence in the future. The caveat with this approach is the risk of inadequate excision similar to other BCS approaches. If there is uncertainty about the extent of disease on pre-operative imaging, staged approach of reconstruction with CWPF could be considered [40, 41]. This involves performing a wide local excision and saline fill of the cavity, followed by second stage reconstruction after confirming tumour extent and achieving clear margins.

Skin island on the flap is used to reconstruct areola while nipple could be reconstructed either in the immediate or delayed setting. Patients could then have areolar tattooing to improve the aesthetics [56]. Following-up patients with these flaps has not shown any significant flap atrophy after radiotherapy, although the data is limited by short-term follow-up [54, 55].

**Figure 3.**
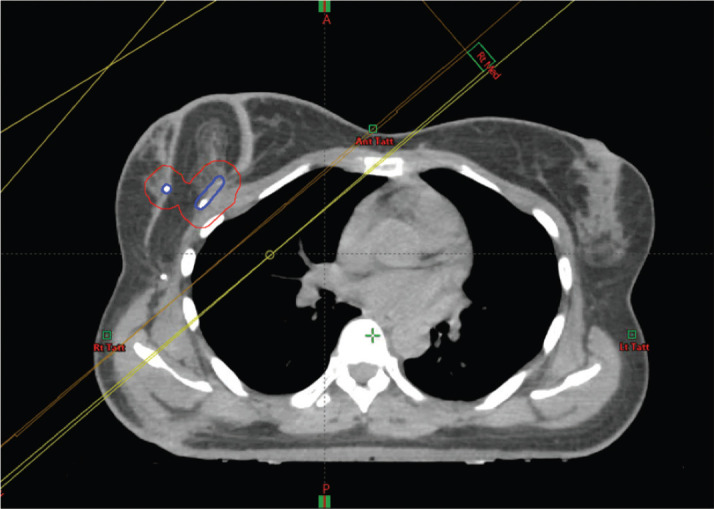
Radiotherapy planning scan of case 3. The tumour bed clips have been identified with the assistance of the surgeon and outlined (in blue) and a 1cm margin added to create a planning target volume (in red).

It is important to assess the impact on radiotherapy planning as some of these reconstruction patterns could add to the complexity of radiotherapy planning. All cases presented in this article had radiotherapy after the surgery. There was no interference reported by oncology colleagues in radiotherapy planning. Our centre practices CT planning for radiotherapy and the flap layout is clearly demonstrated on the cross section (Figure 3). It is, however, important that the tumour bed is marked clearly with clips during surgery to help the radiation oncologist demarcate the area for the tumour bed boost, if needed. If there is any doubt, it is helpful that the oncologist consults their surgical colleague to understand the flap layout and avoid overestimating the area for the tumour bed boost. A recent study shows that a surgeon working together closely with a radiation oncologist can more accurately define the tumour bed boost [57]. The tumour bed clips can at times become displaced by the time of radiation therapy planning [58]. An analysis of 1,933 patients showed that the use of local boost radiation therapy and tumour bed marking was not reported in the majority of studies of oncoplastic BCS [59].

The described approach to reconstruct central breast defect in small to moderate sized non-ptotic breasts using a CWPF offers an additional surgical option in women keen to avoid mastectomy. Patient selection is based on the extent of tumour, size of breast, degree of ptosis and presence of redundant lateral chest wall fold. They should however be warned of mastectomy, if the disease is more extensive than anticipated on initial imaging.

Our case series lacks the patient related outcome measure data, given the small sample size. All patients were pleased to avoid mastectomy and were happy with the aesthetic outcome of the breast as judged between 1 and 2 years after radiotherapy.

## Conclusion

Partial breast reconstruction with CWPF and areolar reconstruction (with or without nipple reconstruction) provides an alternative surgical option for central breast tumours in small to moderate non-ptotic breast. This is the first report described in the literature. Our series have shown excellent aesthetic results in the short-term follow-up period after radiotherapy. This approach obviates the need for mastectomy and potentially for contralateral symmetrisation, as this intervention aims to restore the breast to its pre-treatment size.

## List of abbreviations

CWPF, Chest wall perforator flap; DCIS, Ductal carcinoma in-situ; BCS, Breast conserving surgery; OBS, Oncoplastic breast surgery; LICAP, Lateral intercostal artery perforators; LTAP, Lateral thoracic artery perforator; ER, Oestrogen receptor; PR, Progesterone receptor.

## Compliance with ethical standards

### Statement of human and animal rights

This study was carried out in line with clinical protocols with approval by the local hospital ethics committee (number 4317).

## Informed consent

For this type of study, formal consent is not required.

## Disclosures of potential conflicts of interest

The authors declare that they have no conflicts of interest to disclose.

## Evidence-based medicine

### Level of evidence

Level V, Case Series.

## Funding declaration

No funding received.

## Figures and Tables

**Table 1.** Summary of cases with clinico-pathological details.

	Case 1	Case 2	Case 3	Case 4
Age	50	72	40	36
Co-morbidity	Nil	Hypertension	Hypertension	Nil
Smoking	Nil	Nil	Nil	Nil
Breast size	32B	34C	36C	30AA
Presentation	Screen detected	Symptomatic (lump)	Symptomatic (lump)	Symptomatic (single duct nipple discharge)
Family history	Nil	Nil	Nil	Nil
Radiological size	Bifocal 24 and 15 mm (total extent 54*30 mm)	Unifocal (Mammogram and USS occult), MRI 26 mm)	Unifocal 35 mm	Radiologically occult
Pre-op. histology	Grade 2 IDC with IG DCIS	Grade 2 ILC with HG DCIS	Grade 3 IDC with HG DCIS	Epithelial cells on nipple discharge cytology
IHC	ER8, Her2 negative	ER8, Her2 negative	ER8, Her2 negative	ER8, Her2 negative
Post op. histology	Grade 2 IDC 25 and 16 mm with IG DCIS	Grade 2 ILC 26 and 8 mm with HG DCIS	Grade 3 IDC 35 mm with extensive HG DCIS, separate medial shave had more DCIS < 1 mm to final margin	Grade 1 mucinous 6 mm with HG DCIS on duct excision with margins involving DCIS, G2 mucinous 3 mm with HG DCIS on therapeutic surgery
Lymphovascular invasion	No	No	Yes	No
Sentinel node biopsy	1/1 (macrometastasis, 6 mm, no ECS)	1/2 (macrometastasis, 10 mm, no ECS)	1/1 (micrometastasis, 1 mm, no ECS)	0/1
TNM classification	pT2N1a(sn)	pT2N1a(sn)	pT2N1mi(sn)	pT1bN0(sn)

IDC, Invasive ductal carcinoma, no special type; ILC, Invasive lobular carcinoma; DCIS, Ductal carcinoma in-situ; IG, Intermediate grade; HG, High grade; ECS, Extracapsular spread; USS, Ultrasound scan; IHC, Immunohistochemistry

**Table 2.** Summary of cases with treatment details.

	Case 1	Case 2	Case 3	Case 4
Reason to excise nipple-areola complex	Nipple tethering	Sub-areolar lump	Involved medial margin on vertical scar therapeutic mammoplasty – adjacent to nipple	Incidental cancer on duct excision for nipple discharge
Whole tumour size (in mm)	60	50	60	17 and 6
Further axillary treatment	No further treatment arm of POSNOC trial	Axillary Radiotherapy	Nil	Nil
Specimen weight (in gm)	145	82	170 therapeutic mammoplasty 52 central excision	8.5 total duct excision 35 central excision
Specimen dimensions (ML×AP×SI) mm	86×75×40	77×25×65	80×70×45 40×35×62	45×30×15 55×40×43
Surgical margins	Clear	Inferior margin focally involved with DCIS: re-excised – no further malignancy	NA (No further malignancy seen on central excision)	Clear
Closest peripheral margin	1 mm (superior)	> 5 mm	>5 mm (medial)	10 mm
Duration of surgery (in min)	140	110	120	110
Adjuvant chemotherapy	Yes	No. Oncotype RS 11 (NHS PREDICT 4% for third generation)	Yes, following vertical scar therapeutic mammoplasty	No
Adjuvant radiotherapy	Yes (breast with boost) – POSNOC no axillary RT	Yes (breast, axilla, SCF)	Yes (breast with boost)	Yes (breast)
Adjuvant endocrine therapy	Yes (Anastrozole)	Yes (Anastrozole)	Yes (Tamoxifen). Declined Zoladex, aromatase inhibitor and bisphosphonate	Yes (Tamoxifen)
Wound complication	Nil	Nil	Nil	Nil
Shoulder function recovery	Complete	Complete	Complete	Complete
Aesthetic outcome	Very good	Excellent	Excellent (right bigger than left pre radiotherapy)	Excellent

ML, medio-lateral; AP: antero-posterior; SI, supero-inferior; NA: not applicable; RS, recurrence score; RT, radiotherapy
